# Activation of PAR2 by tissue factor induces the release of the PTEN from MAGI proteins and regulates PTEN and Akt activities

**DOI:** 10.1038/s41598-020-77963-6

**Published:** 2020-12-01

**Authors:** Mohammad A. Mohammad, John Greenman, Anthony Maraveyas, Camille Ettelaie

**Affiliations:** 1 Biomedical Sciences, University of Hull, Cottingham Road, Hull, HU6 7RX UK; 2 Division of Cancer-Hull York Medical School, University of Hull, Cottingham Road, Hull, HU6 7RX UK

**Keywords:** Phosphoinositol signalling, Stress signalling, Mechanisms of disease, Proteases, Blood proteins, Membrane lipids, Tumour-suppressor proteins

## Abstract

Tissue factor (TF) signalling has been associated with alterations in Akt activity influencing cellular survival and proliferation. TF is also shown to induce signalling through activation of the protease activated receptor (PAR)2. Seven cell lines were exposed to recombinant-TF (rec-TF), or activated using a PAR2-agonist peptide and the phosphorylation state of PTEN, and the activities of PTEN and Akt measured. Furthermore, by measuring the association of PTEN with MAGI proteins a mechanism for the induction of signalling by TF was proposed. Short term treatment of cells resulted in de-phosphorylation of PTEN, increased lipid-phosphatase activity and reduced Akt kinase activity in most of the cell lines examined. In contrast, continuous exposure to rec-TF up to 14 days, resulted in lower PTEN antigen levels, enhanced Akt activity and increased rate of cell proliferation. To explore the mechanism of activation of PTEN by TF, the association of "membrane-associated guanylate kinase-with inverted configuration" (MAGI)1–3 proteins with PTEN was assessed using the proximity ligation assay and by co-immunoprecipitation. The interaction of PTEN with all three MAGI proteins was transiently reduced following PAR2 activation and explains the changes in PTEN activity. Our data is first to show that PAR2 activation directly, or through exposure of cells to TF releases PTEN from MAGI proteins and is concurrent with increases in PTEN phosphatase activity. However, prolonged exposure to TF results in the reduction in PTEN antigen with concurrent increase in Akt activity which may explain the aberrant cell survival, proliferation and invasion associated with TF during chronic diseases.

## Introduction

Tissue factor (TF) initiates the coagulation mechanism through formation of a complex with factor VIIa (fVIIa) which then activates factors X and IX^1,2^. TF is present on the surface of cells and is also released within cell-derived microvesicles^3–6^. In addition to its procoagulant function, TF possesses signalling properties both in the cells expressing the protein, as well as on exposure of the recipient cells to exogenous TF-containing microvesicles^7,8^. TF has been strongly associated with more aggressive cancer types and the link between TF and cellular survival, proliferation and migration has been established^9,10^. A number of studies have reported the association of the Akt pathway with TF expression and/or the treatment of cells with fVIIa (or fVIIai)^11–13^ in cells which already express TF^11–16^. Enhanced Akt activation following the incubation of TF-positive cells with fVIIa requires the proteolytic activity of fVIIa^11–15^. However, differing reports attribute Akt activation to be both dependent^16–18^ and independent of protease activated receptor (PAR) 2 signalling^19,20^. It has also been shown that fVIIa signalling suppresses Akt phosphorylation in a TF-cytoplasmic domain dependent manner^18^. Furthermore, work carried out in our laboratory^21^ and reported by Aharon et al.^7^ has demonstrated that acute exposure of cells to TF, or inability to release excess TF^22,23^ can induce cellular apoptosis. In addition, PAR2 signalling has been reported to suppress^18^ or alternatively enhance PI3K/Akt activation^16,17^ while conversely, Akt is reported to interfere with PAR2 signalling^24^.

Phosphatase and tensin homolog (PTEN) is a protein- and lipid-phosphatase which acts as one of the key regulators of the PI3K-Akt pathway and has been identified as a tumour suppressor. The loss of PTEN through mutational inactivation has been strongly associated with many cancers^25–28^. These alterations have been identified as markers of the severity of the progression of cancer^29–32^, as well as the aberrant formation of tissue and tumourgenesis^33,34^. However, reductions in the levels of cellular PTEN are also known to alter the progression of a number of cancers and are detrimental in the pre-cancerous growth and tumourgenesis. Furthermore, mutational loss of the PTEN gene not only elevates the probability of carcinogenesis, but also has been associated with disorders including Cowden syndrome and Bannayan-Riley-Ruvalcaba syndrome which are characterised by the development of non-cancerous tumours^35–37^. The impairment in PTEN activity due to either functional mutation or deletion has been reported to promote tumourgenesis in breast^38^, renal^39^, prostate^40^, head and neck^41^ and lung cells^42^. Therefore, the non-mutagenic deregulation of PTEN is likely to be an important linkage between chronic inflammation and tumourgenesis. PTEN suppresses Akt activity by converting PI(3,4,5)P_3_ to PI(4,5)P_2_, preventing the localisation of Akt to the inner side of the plasma membrane^43–45^. Consequently, PTEN has been classified as a key tumour-suppressor and the loss of PTEN is known to significantly influence cancer progression^29,46^. The activity of PTEN is regulated through de-phosphorylation^47,48^ coupled with recruitment to the cell membrane which in turn enhances its lipid-phosphatase function^49,50^. It has also been reported that the recruitment and activation of PTEN to the membrane is concurrent with binding to membrane-associated guanylate kinase with inverted configuration (MAGI) proteins^51–54^.

To date, four MAGI proteins have been identified (MAGI1-3 and MAGIX). MAGI1-3 have been reported to be capable of binding PTEN^51–54^ but at present, no evidence for the interaction between PTEN and MAGIX exists. The MAGI proteins contain a number of separate protein binding motifs including WW and PDZ domains, with the latter being involved in binding of MAGI to c-terminus of PTEN^54^. MAGI proteins have been implicated in the control of cell migration and invasion through altering the activity of PTEN and modulating Akt signalling^51–54^. Moreover, it is known that the de-phosphorylation of PTEN also accelerates its degradation and can therefore moderate the total amount of PTEN within the cell^53^.

In the current study, the influence of acute and prolonged exposure of a panel of seven cancer cell lines to exogenous recombinant TF, as well as PAR2 activation, on both PTEN and Akt activities, and cell proliferation was examined. In addition, a novel mechanism for the observed outcomes, involving MAGI proteins has been proposed.

## Material and methods

### Cell culture, cellular activation and determination of cell numbers

Seven cell lines were selected to include a range of TF and PAR2 expression^55^ and not on the basis of tissue of origin. Cells lines (ATCC, Teddington, UK) MDA-MB-231 (breast cancer) and PANC-1 (pancreatic cancer) cells were cultured in DMEM; AsPC-1 (pancreatic cancer) and T47-D (breast cancer) lines were cultured in RPMI-1640; MCF-7 (breast cancer) and CaCo-2 (colorectal cancer) cells were cultured in EMEM and LoVo (colorectal cancer) cells were cultured in Ham’s F-12K medium. All media were obtained from Lonza (Cambridge, UK) and contained foetal calf serum 10% (v/v). Cells (2 × 10^5^) were seeded out into 12-well plates and activated either by the addition of recombinant TF (0–1300 pg/ml; Dade Behring, Deerfield, USA) or by incubation with PAR2-agonist peptide (PAR2-AP; SLIGKV; 20 µM) and incubated for durations stated in the results section. In some experiments selected cell lines were pre-incubated with a blocking antibody against PAR2 (SAM-11; 20 µg/ml; Santa Cruz Biotechnology Heidelberg, Germany). The reagents were previously determined to be free of endotoxin using the Limulus Amebocyte Lysate kit (LAL, Cambrex Bio Science, Wokingham, UK)^56^. This particular commercial recombinant TF was used as an activating agent, because it is capable of acting as a co-factor for fVIIa for activating PAR2, but it lacks the cytoplasmic domain. Cell numbers were determined by crystal violet staining using a kit obtained from Active Motif (La Hulpe, Belgium) as previously described and confirmed^22,57^. The cell numbers were interpreted from separate standard curve constructed for each cell line.

### SDS-PAGE and western blot analysis

Cells were lysed in Laemmli's buffer and were separated by 12% (w/v) SDS-PAGE^58^, transferred onto nitrocellulose membranes and then blocked with TBST (10 mM Tris–HCl pH 7.4, 150 mM NaCl, 0.05% Tween-20). Western blot analysis of PTEN phosphorylation in the samples was carried out using a rabbit anti-human phosphoSer380/Thr382/Thr383-PTEN (Cell Signalling Technologies/New England Biolabs, Hitchin, UK) diluted 1:2000 (v/v) in TBST. Total PTEN was detected using a polyclonal rabbit anti-human PTEN antibody (Cell Signalling) diluted 1:2000 (v/v) in TBST. GAPDH was detected using a rabbit anti-human GAPDH polyclonal antibody (Santa Cruz Biotechnology, Heidelberg, Germany), diluted 1:4000 (v/v) in TBST. The membranes were then washed with TBST and probed with a goat anti-rabbit alkaline phosphatase-conjugated antibody (Santa Cruz), diluted 1:4000 (v/v). Bands were then visualised using the Western Blue stabilised alkaline phosphatase-substrate (Promega Corp., Southampton, UK), recorded and analysed using ImageJ program. On each occasion western blot membrane was cut into two and the higher molecular weight section was probed for PTEN or pPTEN, while the lower section was probed for GADPH. The numbers were normalised on all occasions.

### Determination of the relative amount of PTEN mRNA by RT-PCR

Total RNA was isolated using the TRI-reagent system (Sigma Chemical Company, Poole, UK) from 2 × 10^5^ cells and 100 ng of total RNA was used for each reaction. The relative amounts of PTEN mRNA was determined using QuantiTect primer sets to detect PTEN and β-actin (Qiagen, Manchester, UK). The reaction was carried out at an annealing temperature of 60 °C for 1 min using the GoTaq 1-Step RT-qPCR System (Promega Corporation Ltd, Southampton, UK) on an iCycler thermal cycler (Bio-Rad, Hemel Hempstead, UK) for 40 cycles. Following amplification, the relative amounts of PTEN mRNA were determined using the 2^−ΔΔCT^ method and PTEN:β-actin ratios were calculated.

### Determination of PTEN antigen, PTEN lipid-phosphatase activity and Akt kinase activity using ELISA-based assay kits

Cells (2 × 10^5^) were seeded out into 12-well plates and treated with rec-TF or PAR2-AP as described above. The cells were then lysed in PhosphoSafe buffer (150 µl; Merck-Millipore, Watford, UK) containing 1% (v/v) protease inhibitor cocktail (Sigma). The protein concentration in the lysates was determined using the Bradford protein estimation assay and the amount of cellular PTEN was measured in lysates (adjusted to 50 µl) using the Human PTEN ELISA kit (Abcam, Cambridge, UK). The ability of PTEN to hydrolyse PIP3 to PIP2 was measured in cell lysates (6 µl) using a PTEN Activity ELISA kit (echelon Biosciences/LuBioScience, Zurich, Switzerland). The activity of Akt was measured in the samples (30 µl) using the Akt Kinase activity assay kit (Enzo Life Sciences, Exeter, UK) according to manufacturer's instructions.

### Duolink proximity ligation (PLA) assay

The procedure was carried out using the Duolink reagents (Sigma) and adapted from that previously described in detail^59^ with minor modifications. MDA-MB-231 cells (10^3^) were seeded out into 35 mm-glass based μ-dishes (InVitro Scientific/Cellvis, Sunnyvale, USA) and adapted to serum-free medium for 1 h prior to activation. Sets of cells were then incubated with PAR2-AP (20 μM) for up to 30 min and then fixed with 4% (v/v) paraformaldehyde for 15 min. The cells were then washed three times with PBS and permeabilised with Triton X-100 0.1% (v/v) in PBS, for 5 min. All samples were blocked with Duolink blocking buffer for 1 h and incubated overnight with combinations of antibodies as follows, at 4 °C. To examine the potential interactions between PTEN and MAGI1-3, a mouse anti-PTEN antibody (217702; 1 μg/ml; R&D Systems) was used together with rabbit anti-MAGI1 antibody (H-70; 2 μg/ml; Santa Cruz), rabbit anti-MAGI2 antibody (C3; 1 μg/ml; GenTex/Insight Biotechnologies, Wembley, UK) and rabbit anti-MAGI3 antibody (1 μg/ml; Novus/R&D Systems, Abingdon, UK). As controls, the antibodies were substituted with rabbit or mouse IgG isotypes (New England Biolabs; 2 μg/ml and 1 µg/ml respectively). Alternatively, the secondary antibodies (probes) were in turn omitted and the assay carried out to ensure the specificity (Supplementary Fig. 1). The cells were washed three times with PBS and PLA performed according to the manufacturer’s instructions. The cells were stained with DAPI (2 μg/ml) and Phalloidin-FITC (2 µg/ml). Images were acquired using a Zeiss Axio Vert.A1 inverted fluorescence microscope with a × 40 magnification (Carl Zeiss Ltd, Welwyn Garden City, UK). The number of red fluorescent events and nuclei were determined using ImageJ, in 10 fields of view from each assay.

### Co-immunoprecipitation of proteins

Cells were lysed in PhosphoSafe Extraction Reagent (Sigma) containing protease inhibitor cocktail and cell debris removed by centrifugation. MAGI2 protein was immunoprecipitated from cell lysates using the anti-MAGI2 (C3; 4 µg) antibody. To ensure specificity, a rabbit IgG isotype (4 µg) was also included as well as an additional control without any antibody. The samples were incubated at 4 °C overnight with gentle shaking. Pureproteome protein A-magnetic beads (10 µl) (Merck-Millipore) was added to all samples and controls and incubated at 4 °C for 90 min^60^. The tubes were then placed in a magnetic stand and the supernatant removed, washed five times with PBST (1 ml) and the samples denatured in SDS-PAGE loading buffer (70 µl) (Sigma). Samples were then separated by SDS-PAGE, transferred to nitrocellulose membranes and blocked. The membranes were probed with a mouse anti-PTEN antibody (217702), as well as a rabbit anti-MAGI2 antibody and then developed as described above.

### Statistical analysis

All data represent the calculated mean values from the number of experiments stated in each figure legend ± the calculated standard error of the mean. Statistical analysis was carried out using the Statistical Package for the Social Sciences (SPSS Inc. Chicago, USA). Significance was determined using one-way ANOVA (analysis of variance) and Tukey’s honesty significance test or where appropriate, by paired t-test.

## Results

### Assessment of the expression of PTEN in the cells lines

**Figure 1:**
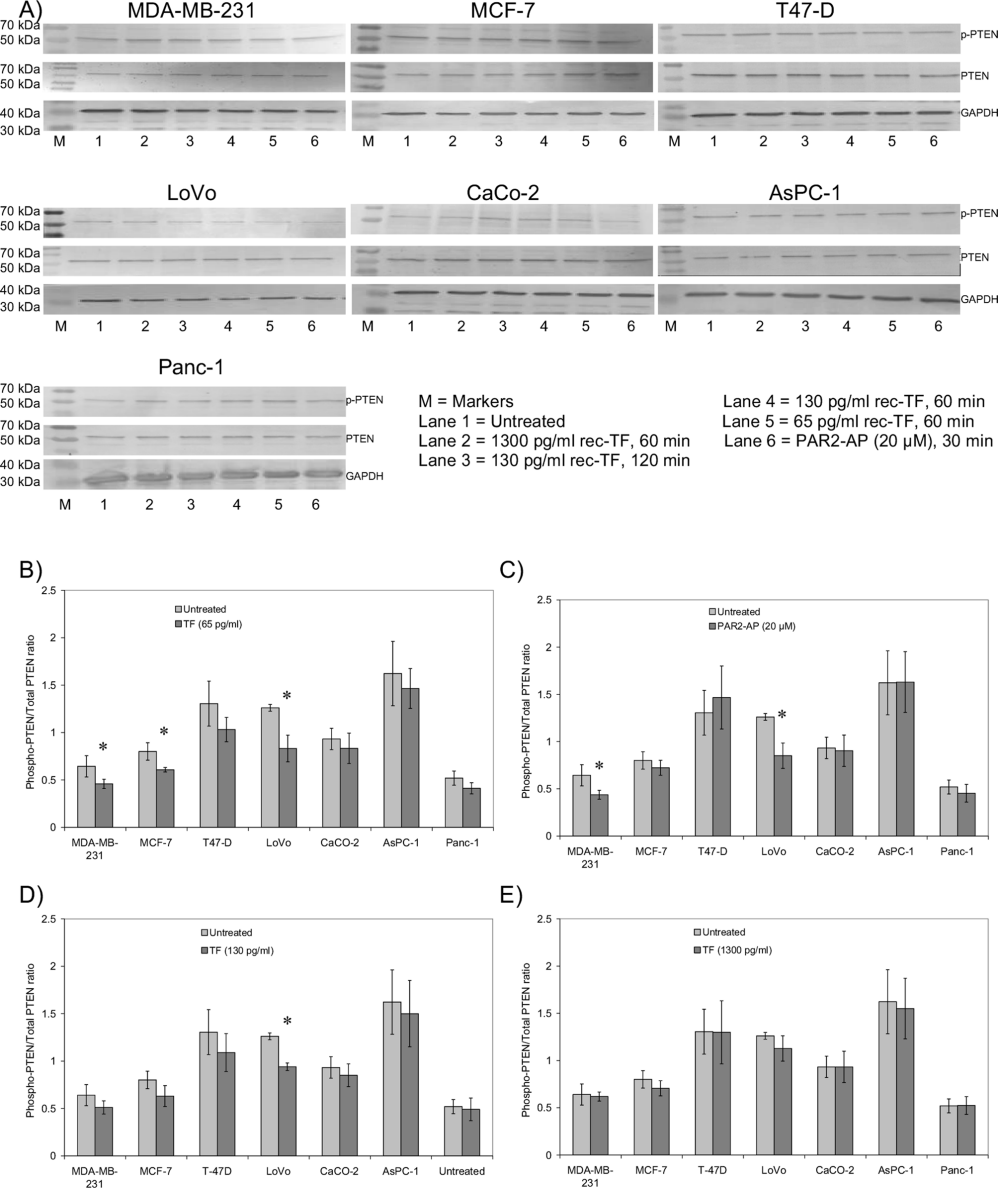
The influence of incubation of cell lines with rec-TF and PAR2-activation on PTEN phosphorylation. (**A**) Cells (MDA-MB-231, MCF-7, T47-D, LoVo, CaCo-2, AsPC-1 and Panc-1) were cultured in the recommended media and activated either by the addition of recombinant TF (0–1300 pg/ml) or by incubation with PAR2-agonist peptide (PAR2-AP; SLIGKV; 20 µM) and incubated for the durations shown. The cells were then lysed in electrophoresis-loading buffer and separated on a 12% (w/v) denaturing polyacrylamide gel. The proteins were then transferred to nitrocellulose membrane and blocked with TBST. The membranes were in turn probed using a rabbit anti-human phosphoSer382/Thr382/Thr383-PTEN, a polyclonal rabbit anti-human PTEN antibody, both diluted 1:2000 (v/v), or a goat anti-human GAPDH polyclonal antibody (V-18), diluted 1:4000 (v/v) in TBST. The membranes were then washed with TBST and probed with goat anti-rabbit or donkey anti-goat alkaline phosphatase-conjugated antibodies as required, diluted 1:4000 (v/v), for 90 min. Bands were then visualised using the Western Blue stabilised alkaline phosphatase-substrate and recorded (micrographs are representative of 6 independent experiments; due to the number of gels the micrographs are cropped to include the main band but also to include at least three marker bands spanning the protein of interest. Full micrograph replicates are included in the supplementary material). The ratio of phospho-PTEN:Total PTEN were determined in the cell samples treated with (**B**) rec-TF (65 pg/ml) for 1 h, and (**C**) PAR2-AP for 30 min (n = 6; *p < 0.05 vs. the respective untreated samples). Similarly, the ratios of phospho-PTEN:Total PTEN were determined in the samples treated with (**D**) rec-TF (130 pg/ml) and (**E**) rec-TF (1300 pg/ml).

While no PTEN deletions/mutations have been reported for any of the cell lines used in this study, this possibility cannot be ruled out. Therefore, in order to exclude the expression of aberrant forms of PTEN in the cell lines, the molecular weight of PTEN was examined by western blot, prior to the study. All the examined cells were shown to express differing amounts of PTEN with an approximate molecular weight of 54 kDa (Fig. 1A, Supplementary Fig. 2), although point mutations of PTEN cannot be ruled out.

### Exposure of cells to TF or PAR2-AP activates PTEN and reduces Akt activity

De-phosphorylation of PTEN at Ser380, Thr382 and Thr383 is a known step in the activation of PTEN^53^. Examination of the phosphorylation state of these amino acids in cell lines showed significant decreases in phospho-PTEN (up to 34%) following a 1 h incubation with rec-TF (65 pg/ml) in MDA-MB-231, MCF-7 and LoVo cells (Fig. 1A,B). Activation of PAR2 in cells resulted in the dephosphorylation of PTEN (up to 32%) in MDA-MB-231 and LoVo cells but was not significant in the other cells tested (Fig. 1A,C). Incubation of cells with 130 pg/ml rec-TF also reduced the phosphorylation of PTEN but was marginally less effective (Fig. 1D) while at 1300 pg/ml no significant influence was detected (Fig. 1E). The lower apparent influence on PTEN dephosphorylation, in response to 1300 pg/ml rec-TF may be as a consequence of immediate irreparable damage to the cells or detachment of the cells since lower remaining cells were available as we have previously indicated^21–23^. Therefore to avoid artefacts, higher concentrations of TF were not used further in this study. The amounts of PTEN protein as determined by the ELISA assay, were not altered by the short-term treatments of the cells (not shown).

**Figure 2:**
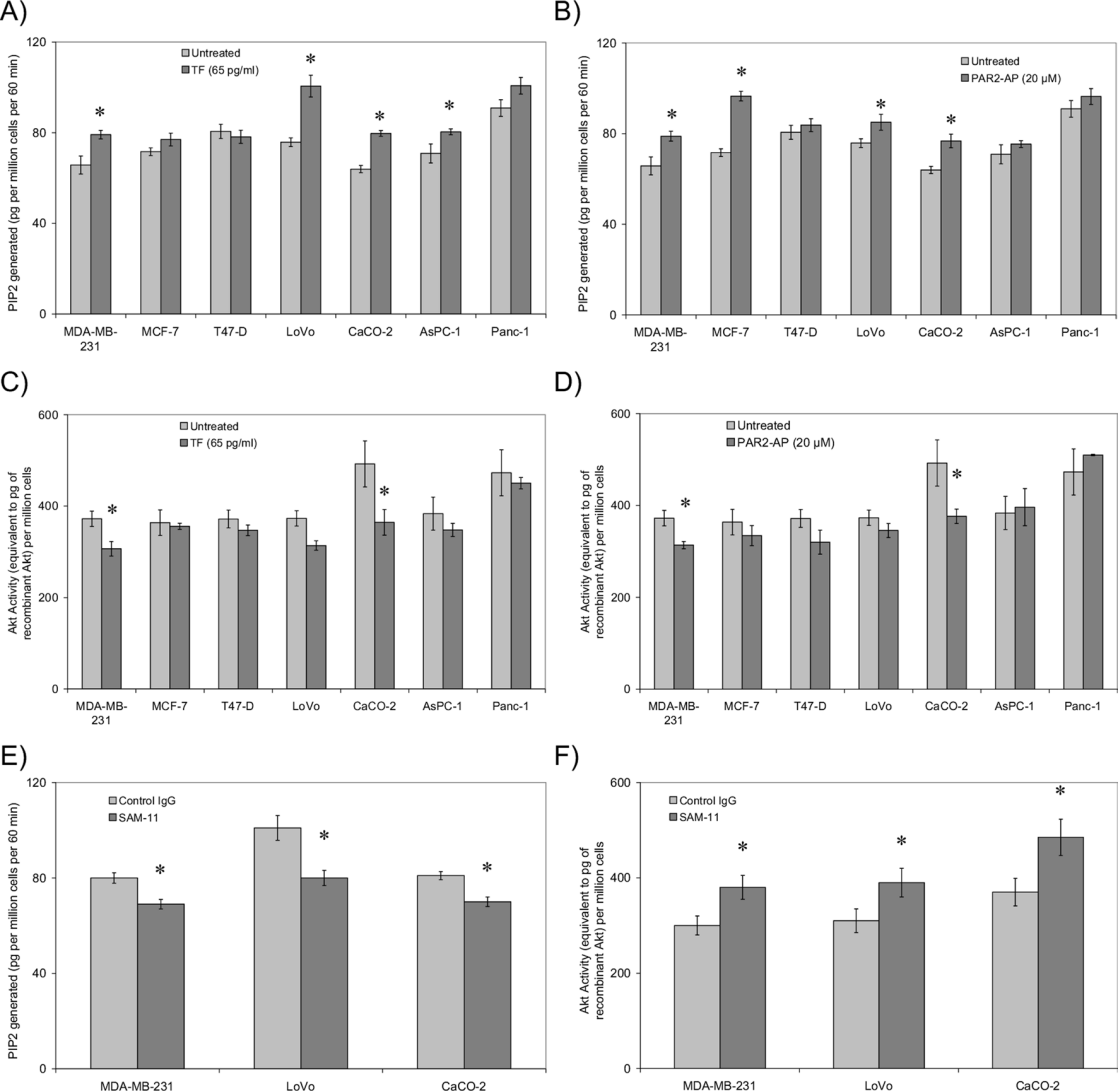
The influence of incubation of cell lines with rec-TF and PAR2-activation on PTEN activity and Akt inhibition. Cells (2 × 10^5^) were treated with (**A**) rec-TF (65 pg/ml) for 1 h, or (**B**) PAR2-AP for 30 min, lysed in PhosphoSafe buffer (150 µl) and the lipid-phosphatase activity of PTEN measured using the echelon PTEN Activity ELISA kit (n = 3; *p < 0.05 vs. the respective untreated samples). Akt activity was also determined in the samples treated with (**C**) rec-TF or (**D**) PAR2-AP as above, and measured using the Akt Kinase activity assay kit according to manufacturer's instructions (n = 3; *p < 0.05 vs. the respective untreated samples). Samples of MDA-MB-231, LoVo and CaCo-2 cells were pre-incubated with a PAR2 blocking antibody (SAM-11, 20 µg/ml) or a control isotype antibody. The cells were treated with recombinant TF and (**E**) PTEN activity and (**F**) Akt activity measured as above (n = 3; *p < 0.05 vs. the respective control isotype antibody).

PTEN is currently the only established phosphatase capable of hydrolysing Phosphatidyl-Inositol(3,4,5) triphosphate (PIP3) to Phosphatidyl-Inositol(4,5) triphosphate (PI4,5P2)^61–65^. However, to ensure the specificity of the PTEN activity kit used in this study, the PTEN antibody was used to immune-deplete a sample of cell lysate along with a control which was immunoprecipitated using an isotype antibody. The PTEN activities of the two samples were then examined using the assay. Comparison of the PTEN activities indicated the total removal of PI4,5P2 formation potential and therefore confirmed the specificity of the assay kit (Supplementary Fig. 3). Measurement of lipid phosphatase activity of PTEN indicated a general increase (up to 33%) in response to rec-TF with MDA-MB-231, LoVo and CaCo-2 cells exhibiting the highest increases compared to untreated cells (Fig. 2A). PTEN activity was also enhanced in MDA-MB-231, MCF-7 and CaCo-2 cells following PAR2 activation (up to 34%) (Fig. 2B). These increases in PTEN activity inversely correlated with reductions in Akt kinase activity (Pearson correlation = − 0.945; p = 0.003). Akt activity decreased in MDA-MB-231 and CaCo-2 cells by up to 26% following incubation with rec-TF and up to 23% in response to PAR2 activation (Fig. 2C,D). Incubation of cells with rec-TF did not alter the PTEN or Akt activity when measured at earlier time-points. Since MDA-MB-231, LoVo and CaCo-2 were found to be most responsive, these cell lines were selected for additional studies as outlined below. Both the increase in PTEN activity and the reduction in Akt activity were dependent on the activation of PAR2 since the pre-incubation of selected cell lines (MDA-MB-231, LoVo and CaCo-2) with a blocking antibody against PAR2 (SAM-11) prevented the effects of additions of TF (Fig. 2E,F).

### Prolonged exposure of cells to rec-TF increases cell proliferation

**Figure 3:**
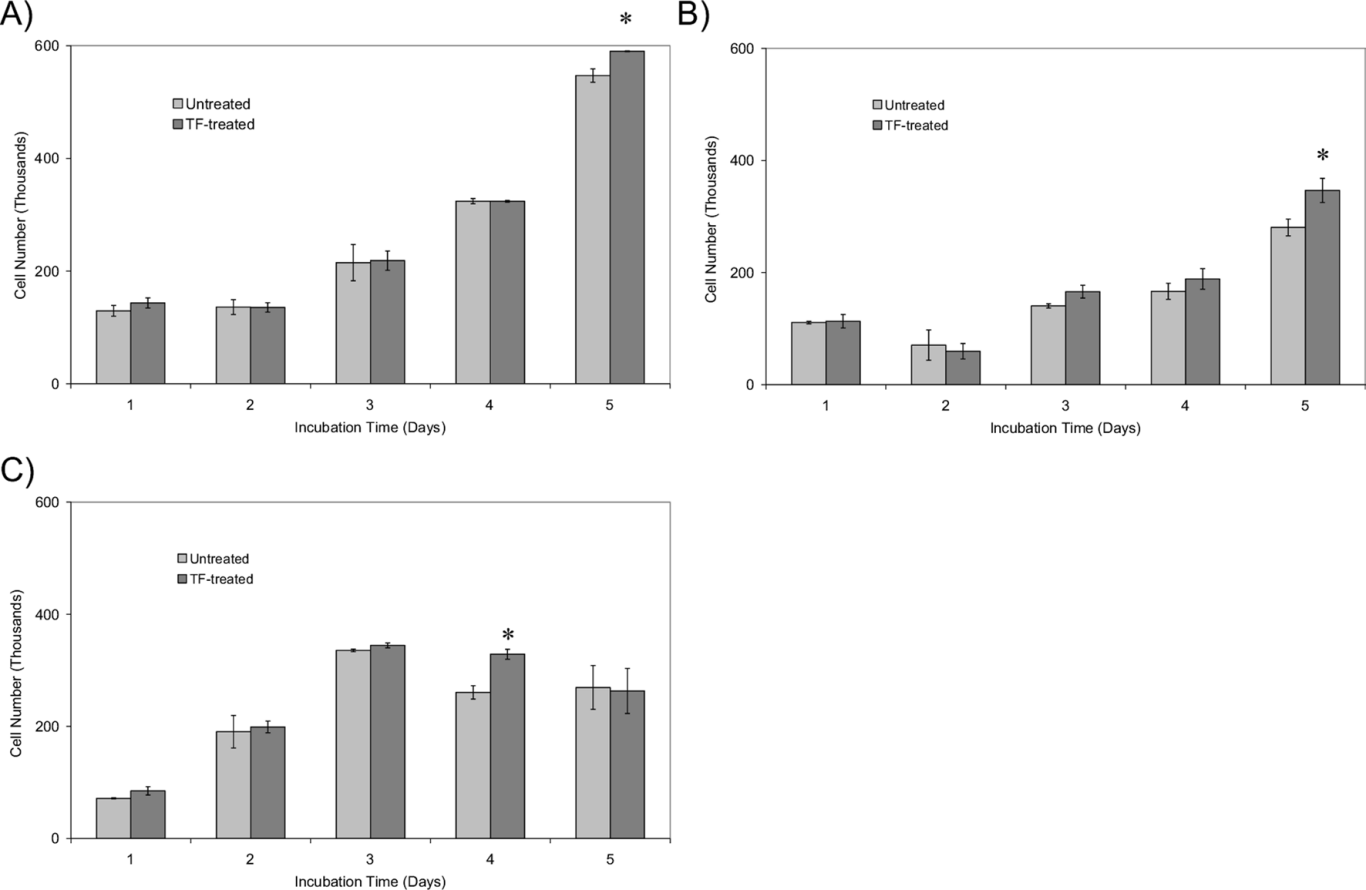
The influence of long-term treatment of cell lines with rec-TF on cell numbers. Cells (2 × 10^5^) were cultured in the recommended media and supplemented every 2 days with rec-TF (65 pg/ml). The number of cells in the treated and untreated samples were determined using crystal violet staining (n = 3; *p < 0.05 vs. the samples on first day 0) in (**A**) MDA-MB-231, (**B**) LoVo and (**C**) CaCo-2 cell lines.

**Figure 4:**
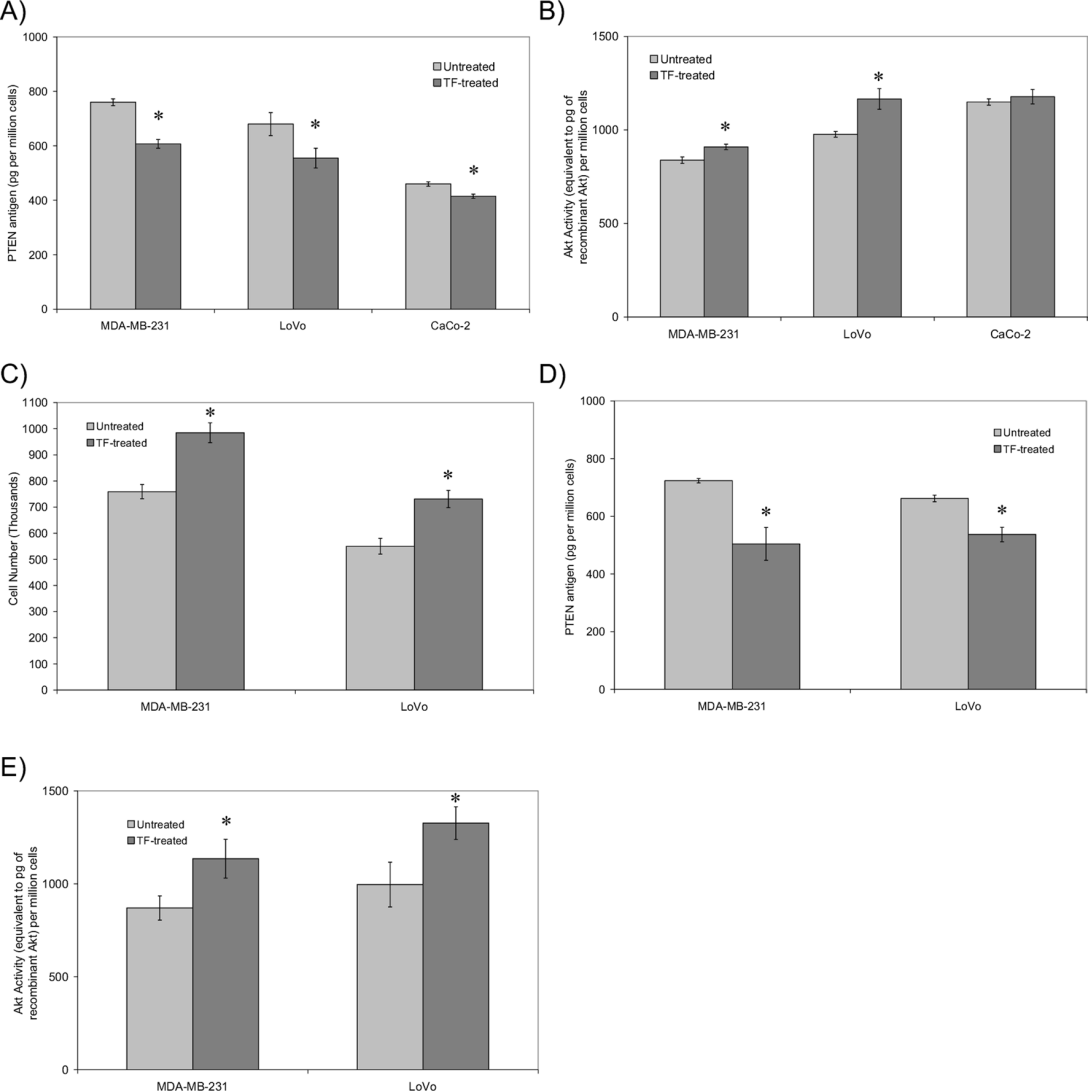
The influence of long-term treatment of cell lines with rec-TF on cellular PTEN antigen levels and Akt activity. Equal number of cells were lysed in PhosphoSafe buffer (150 µl) and (**A**) the level of PTEN antigen measured using a PTEN ELISA kit (n = 5; *p < 0.05 vs. the samples on first day 0). (**B**) The Akt activity was also measured using the Akt Kinase activity assay kit (n = 3; *p < 0.05 vs. the samples on first day 0). Cells (2 × 10^5^) were cultured in the recommended media and supplemented every 2 days with rec-TF (65 pg/ml) up to 14 days. (**C**) The number of cells in the treated and untreated samples were determined using crystal violet staining (n = 3; *p < 0.05 vs. the samples on first day 0). In addition, cells were lysed in PhosphoSafe buffer and (**D**) the level of PTEN antigen and (**E**) the Akt kinase activity measured. (n = 3; *p < 0.05 vs. the samples on first day 0).

To achieve the sustained activation of the cells, prolonged incubation of cells was carried out by supplementing the cells with rec-TF (65 pg/ml) on day 0, 2 and 4. This treatment of cells resulted in increased cell proliferation by day 5 in MDA-MB-231 (10%), LoVo cells (24%) (Fig. 3A,B) and by day 4 in CaCo-2 cells (26%; Fig. 3C). Furthermore, exposure of cells to rec-TF reduced the level of cellular PTEN antigen in MDA-MB-231 (20%), LoVo (18%) and CaCo-2 (10%) cells by the fifth day, compared to equivalent untreated samples (Fig. 4A). However, PTEN mRNA levels remained constant throughout the treatments (Supplementary Figure 4). Prolonged exposure of cells to rec-TF also resulted in the enhancement of Akt activity in MDA-MB-231 (9%) and LoVo (29%) but was not significant in CaCo-2 cells (Fig. 4B). In a similar set of experiments, the MDA-MB-231 and LoVo cells were treated with rec-TF for 14 days. The increases in cell numbers were determined to be 30% and 33% (Fig. 4C), while the level of cellular PTEN declined by 30% and 23% respectively (Fig. 4D) compared to untreated cells. However, the enhancements in Akt activity remained at 13% and 29% above untreated cells respectively (Fig. 4E) suggesting that such small but persistent increases in Akt activity may become a significant factor in the cumulative divergence in the rate of cell proliferation, such as those observed in some chronic conditions.

### Activation of PAR2 releases PTEN from MAGI complexes

For the subsequent experiments, MDA-MB-231 and LoVo cells were considered as suitable cell lines for examining the association of PTEN with MAGI1-3, using the proximity ligation assay (PLA). However, because of their smaller size and also complications arising from cell stacking, the analysis of LoVo cells was found to be erroneous and consequently, further studies were carried out in MDA-MB-231 cells only.

**Figure 5:**
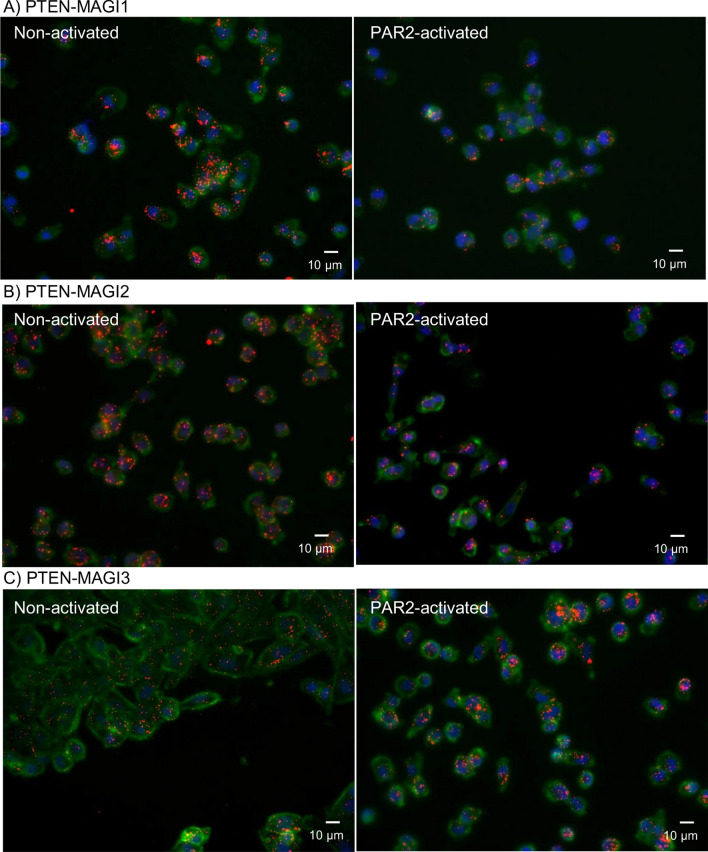
Analysis of the proximity of PTEN and MAGI1-3 by proximity ligation assay and the influence of PAR2 activation. MDA-MB-231 cells (10^3^) were seeded out into 35 mm-glass based μ-dishes and adapted to serum-free medium for 1 h prior to activation. The cells were then incubated with PAR2-AP (20 μM) for up to 30 min. The proximity between PTEN and MAGI1-3 were examined using a mouse anti-human PTEN (217702) diluted 1:100 (v/v) together with a rabbit anti-MAGI1 antibody (H-70; 2 μg/ml), a rabbit anti-MAGI2 antibody (1 μg/ml) and a rabbit anti-MAGI3 antibody (1 μg/ml). The cells were then labelled with DAPI (2 μg/ml) and Phalloidin-FITC (2 µg/ml). Images were acquired using a Ziess Axio Vert.A1 inverted fluorescence microscope with a ×40 magnification (the micrographs are representative of 10 fields of view from 4 independent experiments RED = PLA incidences; GREEN = Phalloidin; BLUE = DAPI).

**Figure 6:**
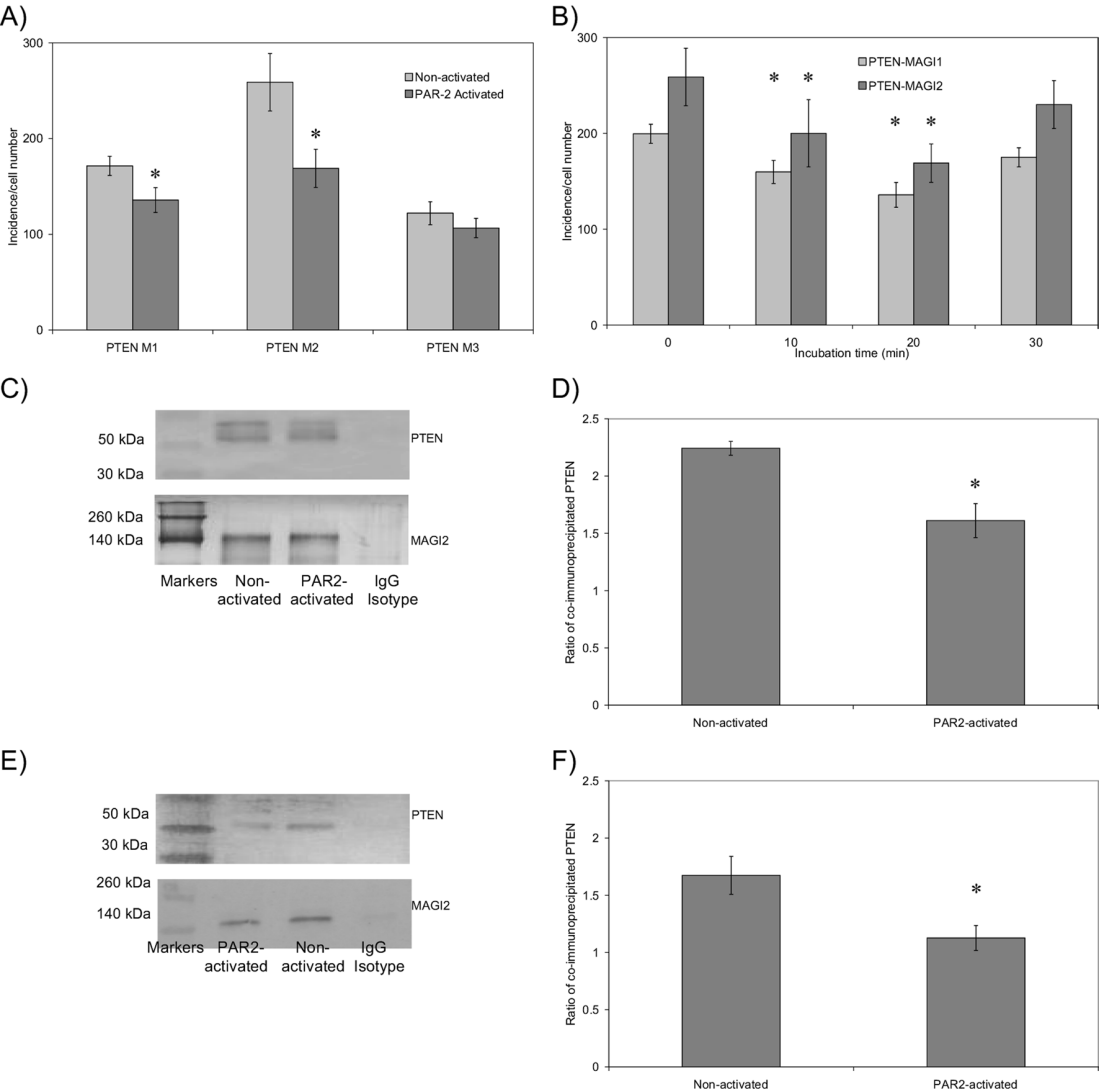
Analysis of the interaction of PTEN and MAGI1-3 and the influence of PAR2 activation. MDA-MB-231 (10^3^) were seeded out into 35 mm-glass based μ-dishes and adapted to serum-free medium for 1 h prior to activation. The cells were then incubated with PAR2-AP (20 μM) for up to 30 min and analysed by PLA as described in Fig. 5. The number of red fluorescent events and nuclei were determined using ImageJ, in 10 fields of view from each assay for (**A**) the interactions of PTEN with MAGI1-3 in non-activated and at 20 min post-activation. (**B**) In addition, the interaction of PTEN and MAGI1 and 2, was measured at intervals up to 30 min (n = 3; *p < 0.05 vs. the non-activated sample). MAGI2 was immunoprecipitated from cell lysates with an anti-MAGI2 (C3; 4 µg) antibody using protein A-magnetic beads. The MDA-MB-231 lysate samples were washed five times with PBST (1 ml) and denatured in SDS-PAGE loading buffer and (**C**) examined for PTEN and MAGI2 by western blot using a mouse anti-PTEN antibody (217702) and a rabbit anti-MAGI2 antibody. (**D**) The ratio of the PTEN band densities were normalised against those of MAGI2 in the same co-immunprecipitated samples (n = 3; *p < 0.05 vs. the non-activated sample). Similarly, the LoVo lysate samples were used to immunoprecipitate MAGI2 and (**E**) examined for PTEN and MAGI2 by western blot using a mouse anti-PTEN antibody (217702) and a rabbit anti-MAGI2 antibody. (**F**) The ratio of the PTEN band densities were normalised against those of MAGI2 in the same co-immunprecipitated samples (n = 3; *p < 0.05 vs. the non-activated sample). Full micrograph replicates are included in the supplementary material.

Since PTEN has been reported to interact with all three MAGI proteins^25,53,66^, the association of PTEN with the MAGI proteins was examined. Analysis of the non-activated cells using PLA suggest that PTEN is mainly associated with MAGI2 and the least amount with MAGI3 (Figs. 5 and 6A). Moreover, the activation of cells using PAR2-AP resulted in 22% and 35% reduction in the association of PTEN with MAGI1 and MAGI2 respectively, but no significant change was observed with MAGI3 (Fig. 6A). Time-course PLA analysis also showed a transient decrease in the association of PTEN with MAGI1 and MAGI2 by 20 min post-activation with PAR2-AP and then was partially restored by 30 min incubation (Fig. 6B). Western blot analysis confirmed the co-immunoprecipitation of PTEN with MAGI2 from non-activated MDA-MB-231 (Fig. 6C,D) and LoVo (Fig. 6E,F) cells which decreased by 28% and 33% following the activation of the two cell lines respectively (full gels are shown in Supplementary Fig. 5). Treatment of cells with PAR2-AP did not alter the amount of cellular MAGI2 in the period of testing (Supplementary Fig. 6).

## Discussion

The signalling properties of TF have been established previously and the ability of the TF to alter the behaviour of cells is well documented. Of particular interest is the ability of TF to confer improved survival in cancer cells, and a notable mechanism has been the activation of Akt pathway following the association of TF with factor VII^11–16^. We hypothesised that the response of cells to acute levels of TF such as those found in injury and trauma, may differ from the adaptive behaviour of the cells that is observed during longer-term chronic inflammation. As discussed above, any deregulation of PTEN is likely to be an important linkage between chronic inflammation and tumourgenesis. For this reason, we monitored PTEN and Akt activities in seven cancer cell lines in the short-term following a single treatment with rec-TF or PAR2-AP, as well as examining the outcome of exposure of cells to repeated doses of TF over longer periods.

The function of PTEN as a tumour suppressor has been well recognised and its ability to downregulate Akt activity through its lipid phosphatase activity has been extensively studied^49–54^. There is already an established association between PTEN mutation/deregulation and increased TF expression in cancer cells^67,68^. It has also been suggested that the tumour cells may express ectopic fVIIa, altering the function of PARs^69–71^. In addition to PAR2 activation, TF may also influence cells directly by interaction with the cell-surface proteins such as β1-integrin^72,73^. In fact, we previously showed the expression of active form fVIIa in MDA-MB-231, MCF-7 and AsPC-1 cells^70,71^ and this constitutes an adequate source of factor fVIIa to drive this mechanism. In these studies, suppression of the expression of fVIIa or PAR2 in MDA-MB-231 cells using verified siRNA, or blocking with respective inhibitory antibodies, disrupted the release of MV and reduced the rate of cell proliferation in otherwise untreated cells. This is in agreement with the findings reported by Åberg et al. who have comprehensively described the increased cell proliferation, through the activation of Akt pathway, following the supplementation of TF-expressing cells with fVIIa^11–13^. In contrast, the outcome of cellular exposure to exogenous TF, and the subsequent activation of PAR2 on PTEN function and PTEN protein levels has not previously been examined. In our experiments the optimal timescale for the activation of PTEN and Akt as well as dissociation of PTEN from MAGI in response PAR2-AP was found to be around 20–30 min and is comparable to other published timescales^74–76^. It is expected that the activation of PAR2 by treatment with recombinant TF would take a similar timescale to that by microvesicles which adds a further 30 min since the TF needs to become incorporated into the cell membrane of cells^77^. To allow sufficient time for the TF to be incorporated and then for the PAR2 signalling to activate PTEN and Akt, we incubated the cells with TF for up to 120 min. However, measurements carried out at 30 min did not reveal any change in the PTEN phosphorylation change, post-treatment with recombinant TF. Our data indicate that either short-term exposure to TF, or the activation of PAR2 can induce some level of PTEN de-phosphorylation in cell lines examined (Fig. 1). In keeping with published studies^47^, de-phosphorylation of PTEN was concurrent with enhanced lipid-phosphatase activity which in our study, was also accompanied by reductions in Akt kinase activity. Following injury or trauma, cells may become exposed to large quantities of TF together with the activation PAR2 by coagulation proteases. Therefore, as a possible explanation, the lowered Akt activity may be a protective mechanism by which the severely damaged cells are prepared for apoptosis and removal, preventing aberrant tissue formation. Moreover, although these effects were significant reaching up to 26% in MDA-MB-231 and CaCo-2 cells, the outcome in vivo, is very likely to involve other deciding inflammatory factors. In agreement with this, cell numbers were shown to be lower after 24 h treatment with TF, compared to the untreated samples in some but not all of the cell lines studied.

Membrane-associated PTEN is stabilised by an interaction with MAGI proteins^78–82^, which is mediated through the c-terminus of PTEN and the second PDZ domain of MAGI proteins^52^. MAGI proteins are mainly located at the cell–cell junctions and are involved in the assembly of junction protein complexes^83^. Examination of MDA-MB-231 cells using PLA indicated the reduced association of PTEN with MAGI proteins in PAR2-activated cells, and was confirmed by co-immunoprecipitation of PTEN with MAGI2 (Figs. 5 and 6). As far as we are aware, the dissociation of PTEN from MAGI proteins in response to PAR2 has not been reported previously. This transient release of PTEN was concurrent with a 20% increase in PTEN activity and 18% reduction in Akt kinase activity when examined in MDA-MB-231 cells (Fig. 2). This is in line with studies comparing the activity of c-terminus truncated PTEN which is incapable of binding to MAGI, to that of the full-length PTEN showing approximately 10% increase in the lipid-phosphatase activity^78^ together with approximately 17% reduction in PI3K activity^79^. Therefore, it is conceivable that the dissociation of PTEN from MAGI may transiently increase the apparent PTEN activity following injury as a response to disruption of cell junctions. Moreover, exogenous TF may form a complex with cell-expressed fVIIa to activate cell-surface PAR2 or alternatively, exogenous TF may interact with cell-surface proteins directly. In agreement with the former hypothesis, rec-TF produced a greater response in cells with higher levels of PAR2, such as LoVo compared to AsPC-1 cells^55^. However, the activation of PAR2 using the agonist peptide did not always produce the same response as addition of rec-TF and therefore, other mechanisms are likely to also be involved. Also, despite the increased PTEN activity, the Akt activity did not significantly change in some cells. Therefore, it is possible that in some cell lines a compromised signalling mechanism prevents the reduction of Akt or a defective regulatory mechanism may artificially maintain the Akt activity at higher levels and therefore, alter the cellular behaviour. This was also true on long-term incubation of CaCo-2 cells with rec-TF in which, despite a reduction in PTEN (Fig. 4A) the Akt activity did not change (Fig. 4B). Therefore, while the signalling pathways arising from PAR2 activation have been characterised, the mechanisms that influence the MAGI proteins need identification and for the present, we have refrained from indicating a correlation between the amount of cell surface PAR2 and the magnitude of PTEN regulation.

The de-phosphorylation of PTEN, especially when not protected by MAGI, can lead to further modification of the PTEN leading to proteosomal degradation of the protein^53^. Therefore, it was envisaged that prolonged exposure of cells to TF may progressively activate and then degrade cellular PTEN protein. Measurement of PTEN antigen levels, in continuously-treated MDA-MB-231 cells showed a reduction in PTEN antigen but not mRNA expression over 5 days, together with increased Akt activity and cell proliferation (Figs. 3 and 4). This depletion of PTEN together with increased Akt activity suggests a mechanism by which the regulation of cell survival, proliferation and apoptosis becomes compromised by prolonged exposure to TF. Low level expression of Akt in mice has been shown to alter the tissue morphology and can compound tumour formation and survival^84^. Therefore, while the increases in Akt activity were significant reaching 29% in LoVo cells, such outcomes are likely to become dominant over longer periods of time as observed during chronic inflammatory diseases. Similarly, it has been reported that subtle reductions in PTEN protein levels increase the susceptibility of cells to tumourgenesis through the activation of the PI3K-Akt pathway^28,85^. Therefore, this study suggests that the prolonged exposure of cells to TF together with the activation of PAR2 may contribute to tumourgenesis through the degradation of PTEN resulting in tumour cell proliferation. Such decreases in PTEN levels have already been shown to have implication in clinical settings, with outcomes influencing non-malignant tumourgenesis^35–37^, carcinogenesis^33,34^ as well as the progression of cancers^29–32^. This study attempted to decipher the molecular and cellular connections between the exposure of TF during inflammatory conditions and the regulation of PTEN and Akt activity and therefore an extended discussion of clinical manifestations and influences remains beyond the remit of this study.

The disruption of cellular layers often occurs as a consequence of injury which and often results in the exposure of TF and the activation of coagulation^86,87^. Under such conditions, it is imperative to make a distinction between the severely injured cells and those which may be revived. Since TF is one of the first proteins which appears at the site of injury, it may possess the ability to subsequently initiate differing signals in order to direct the cells towards apoptosis or proliferation respectively. This study has demonstrated the ability of TF to induce PTEN activation in the short term, while prolonged exposure to TF appears to reduce the total cellular PTEN protein. This may in turn result in aberrant cell survival and proliferation during disease conditions.

## Supplementary information

Supplementary Information.

## Abbreviations

TF: Tissue factor
fVIIa/Xa: Factor VIIa/fXa
PTEN: Phosphatase and tensin homolog protein
PI3K: Phosphoinositol-3-kinase
PIP3: Phosphoinositol-3-phosphate
MAGI: Membrane-associated guanylate kinase-with inverted configuration
PAR2: Protease activated receptor 2
