# Supplementary material for: Activation of PAR2 by tissue factor induces the release of the PTEN from MAGI proteins and regulates PTEN and Akt activities
Source: Sci Rep. 2020 Dec 1;10:20908. doi: 10.1038/s41598-020-77963-6 (PMC7708427; doi:10.1038/s41598-020-77963-6)
Supplement: Supplementary file 1 — Supplementary Information. [file 41598_2020_77963_MOESM1_ESM.docx]

**Activation of PAR2 by tissue factor induces the release of the PTEN from MAGI proteins and regulates PTEN and Akt activities**

Mohammad A Mohammad^1^, John Greenman^1^, Anthony Maraveyas^2^, Camille Ettelaie^1*^

^1^Biomedical Sciences, University of Hull, Cottingham Road, Hull, HU6 7RX, UK. UK, ^2^Division of Cancer-Hull York Medical School, University of Hull, Cottingham Road, Hull, HU6 7RX, UK.

*Correspondence to Dr Camille Ettelaie, Biomedical Section, University of Hull, Cottingham Road, Hull, HU6 7RX, UK

Supplementary Figure 1. Confirmation of lack of cross-reactions for proximity ligation assay


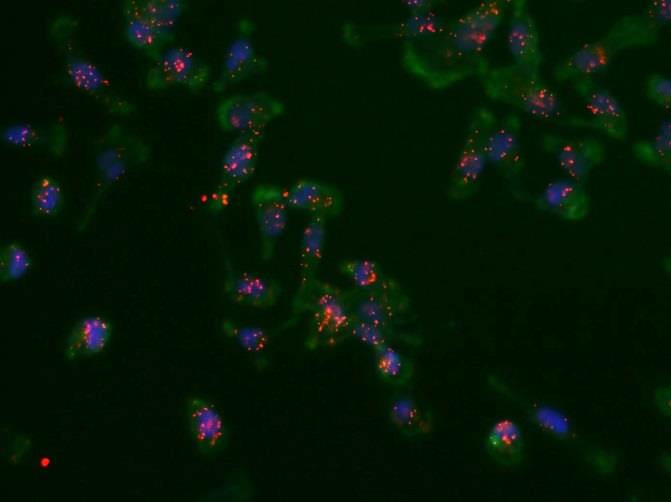

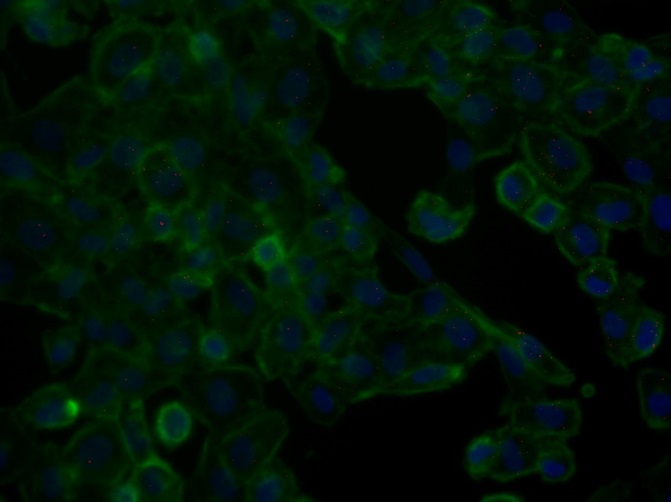


A) Complete antibodies B) No probes

C) No Anti-PTEN antibody D) No Anti-MAGI2 antibody


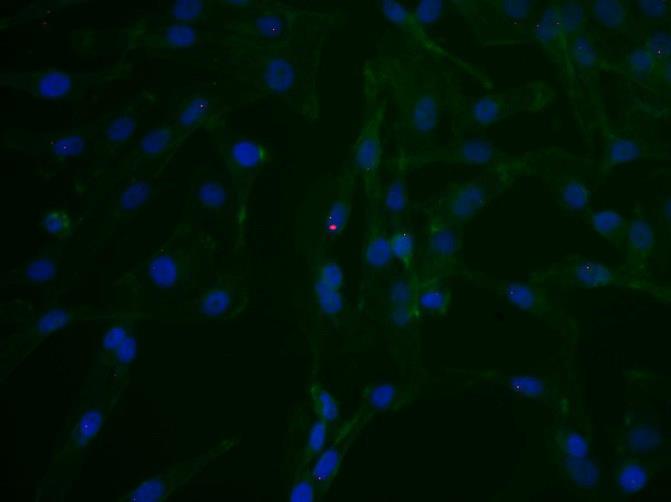

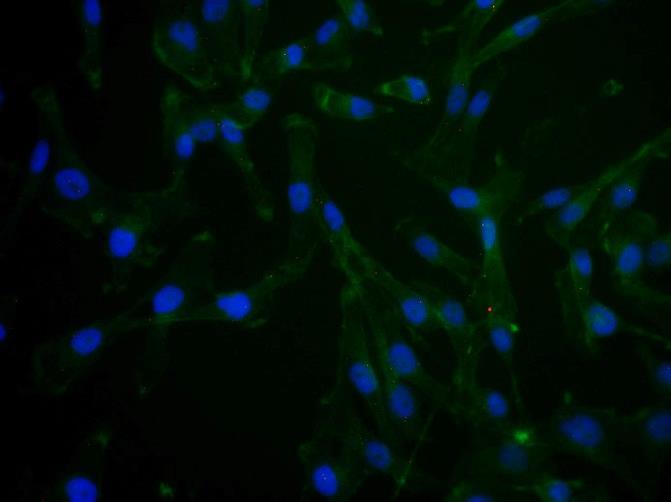


MDA-MB-231 cells (10^3^) were seeded out into 35 mm-glass based μ-dishes and adapted to serum-free medium for 1 h prior to activation. A set of controls were carried out using the proximity ligation assay. A) A mouse anti-human PTEN diluted 1:100 (v/v) was used together with a rabbit anti-MAGI2 antibody (1 μg/ml) and then probed with the positive and negative secondary oligonucleotide-conjugated antibodies. The experiment was repeated but B) omitting the probes, C) without the PTEN antibody and D) in the absence of the MAGI2 antibody. The cells were then labelled with DAPI (2 μg/ml) and Phalloidin-FITC (2 µg/ml). Images were acquired using a Ziess Axio Vert.A1 inverted fluorescence microscope with a ×40 magnification. (The micrographs are representative of 10 fields of view from 4 independent experiments RED= PLA incidences; GREEN = Phalloidin; BLUE = DAPI).

Supplementary Figure 2 Uncompressed micrographs of analysis of PTEB by western blot


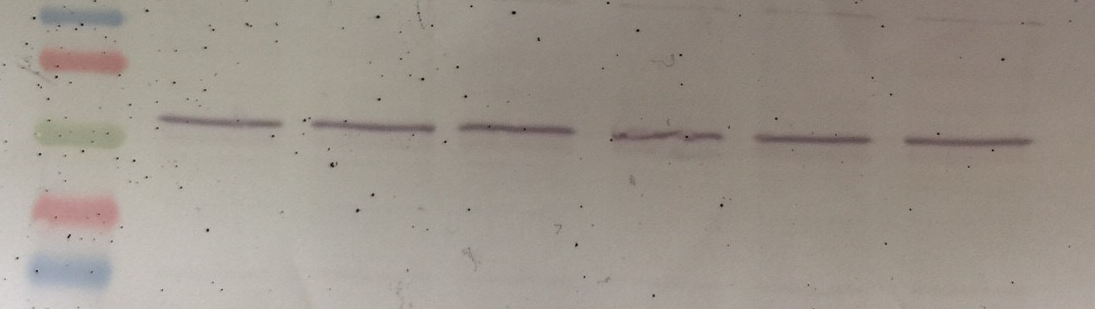


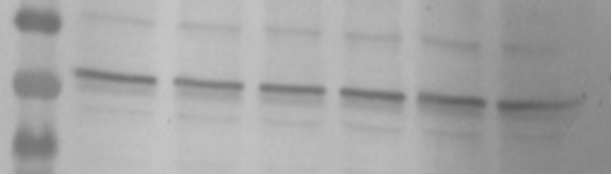
MDA-MB-231

70 kDa

50 kDa

30 kDa

70 kDa

50 kDa

30 kDa


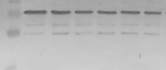
40 kDa

30 kDa

20 kDa

M Lane 1 Lane 2 Lane 3 Lane 4 Lane 5 Lane 6

MCF-7


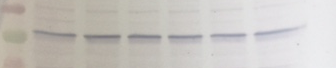


70 kDa

50 kDa

30 kDa

70 kDa
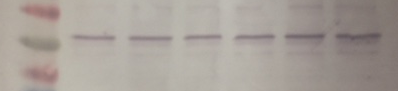


50 kDa

30 kDa


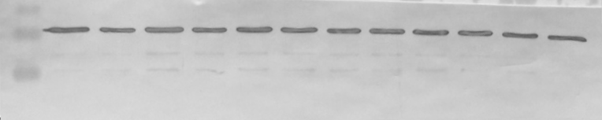


40 kDa

30 kDa

20 kDa

M Lane 1 Lane 2 Lane 3 Lane 4 Lane 5 Lane 6

T47-D


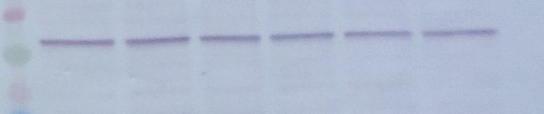


70 kDa

50 kDa

30 kDa


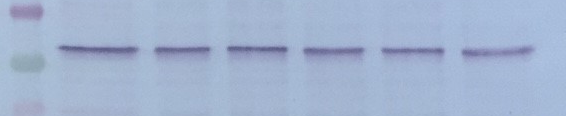


70 kDa

50 kDa

30 kDa


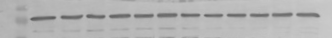


40 kDa

30 kDa

20 kDa

M Lane 1 Lane 2 Lane 3 Lane 4 Lane 5 Lane 6


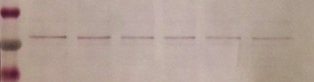
LoVo

70 kDa

50 kDa

30 kDa


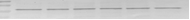


70 kDa

50 kDa

30 kDa


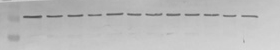


40 kDa

30 kDa

20 kDa

M Lane 1 Lane 2 Lane 3 Lane 4 Lane 5 Lane 6


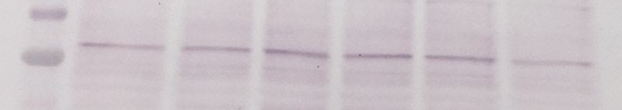
CaCo-2

70 kDa

50 kDa

30 kDa


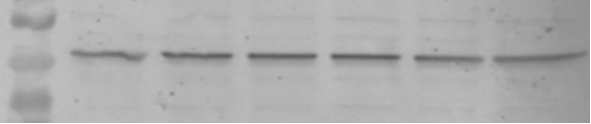


70 kDa

50 kDa

30 kDa


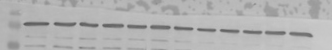


40 kDa

30 kDa

20 kDa

M Lane 1 Lane 2 Lane 3 Lane 4 Lane 5 Lane 6

AsPC-1


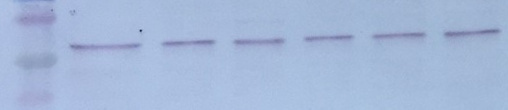


70 kDa

50 kDa

30 kDa


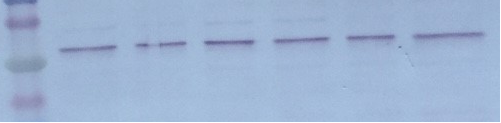


70 kDa

50 kDa

30 kDa


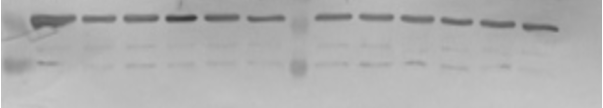
40 kDa

30 kDa

20 kDa

M Lane 1 Lane 2 Lane 3 Lane 4 Lane 5 Lane 6

Panc-1


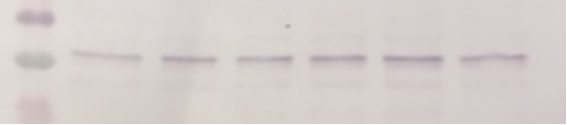


70 kDa

50 kDa

30 kDa


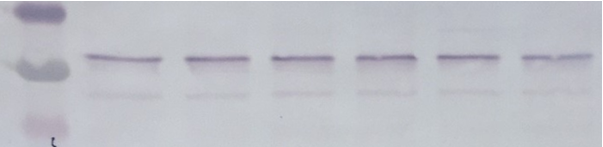


70 kDa

50 kDa

30 kDa


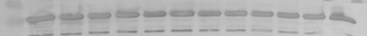


40 kDa

30 kDa

M Lane 1 Lane 2 Lane 3 Lane 4 Lane 5 Lane 6

Cells (MDA-MB-231, MCF-7, T47-D, LoVo, CaCo-2, AsPC-1 and Panc-1) were cultured in the recommended media and activated either by the addition of recombinant TF (0-1300 pg/ml) or by incubation with PAR2-agonist peptide (PAR2-AP; SLIGKV; 20 µM) and incubated for the durations shown. The cells were then lysed in electrophoresis-loading buffer and separated on a 12% (w/v) denaturing polyacrylamide gel. The proteins were then transferred to nitrocellulose membrane and blocked with TBST. The membranes were in turn probed using a rabbit anti-human phosphoSer382/Thr382/Thr383-PTEN, a polyclonal rabbit anti-human PTEN antibody, both diluted 1:2000 (v/v), or a goat anti-human GAPDH polyclonal antibody (V-18), diluted 1:4000 (v/v) in TBST. The membranes were then washed with TBST and probed with goat anti-rabbit or donkey anti-goat alkaline phosphatase-conjugated antibodies as required, diluted 1:4000 (v/v), for 90 min. Bands were then visualised using the Western Blue stabilised alkaline phosphatase-substrate and recorded. (Micrographs are representative of 6 independent experiments; due to the number of gels the micrographs are cropped to include the main band but also to include at least three marker bands spanning the protein of interest). Legend: M = Markers; Lane 1 = Untreated; Lane 2 = 1300 pg/ml rec-TF, 60 min; Lane 3 = 130 pg/ml rec-TF, 120 min; Lane 4 = 130 pg/ml rec-TF, 60 min; Lane 5 = 65 pg/ml rec-TF, 60 min; Lane 6 = PAR2-AP (20 µM), 30 min

Supplementary Figure 3. Examination of PTEN activity in PTEN immune-depleted cell lysate

*

*

MDA-MB-231 cells (10^3^) were seeded out into 35 mm-glass based μ-dishes and adapted to serum-free medium for 1 h prior to activation. Cell lysates were divided into three aliquots and were immune-depleted by incubating with a mouse anti-human PTEN antibody (217702) (20 µg/ml), an IgG isotype (20 µg/ml) or used untreated. All samples were then incubated with a protein A-magentic beads and the analysed along with a control (no sample) using the echelon PTEN assay kit. (n = 3; * = p< 0.05 vs. the no sample)

Supplementary Figure 4. Analysis of PTEN mRNA by qRT-PCR (Table of Ct values)

A)

Cell line Non-treated Rec TF treated

Amplicon PTEN β-actin PTEN β-actin

MDA-MB-231 27.0, 26.6, 26.9 24.0, 23.5, 23.8 27.2, 27.1, 26.9 23.6, 23.9,24.5

LoVo 28.5,28.7,29.1 22.8,23.0,23.4 28.3,29.1,29.0 23.2,23.3,22.9

CaCo2 29.5,29.9.30.1 25.5,24.9,25.3 29.6,29.6,30.1 26.2,25.2,25.8

B)

Cell line Ratio of mRNA (Rec TF treated : Non-treated)

MDA-MB-231 1.00 ± 0.11

LoVo 1.02 ± 0.25

CaCo2 0.98 ± 0.19

Cells were treated with rec-TF (65 pg/ml) or vechicle control for 5 days. Total RNA was isolated using the TRI-reagent system (Sigma Chemical Company, Poole, UK) from 2 × 10^5^ cells and 100 ng of total RNA was used for each reaction. The relative amounts of PTEN mRNA was determined using QuantiTect primer sets to detect PTEN and β-actin. The reaction was carried out at an annealing temperature of 60 °C for 1 min using the GoTaq® 1-Step RT-qPCR System on an iCycler thermal cycler for 40 cycles. A) Ct values were determined and representative values of 3 separate studies are shown. B) Following amplification, the relative amounts of PTEN mRNA were determined using the 2^-ΔΔCT^ method and Ct values for the PTEN and β-actin were used to calculate the ratio of rec-TF treated to non-treated PEN mRNA expression (n = 3 separate experiments each carried out in triplicates).

Supplementary Figure 5. Co-immunoprecipitation of PTEN with MAGI2


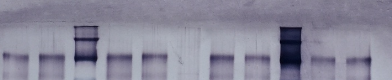


100 kDa

70 kDa

50 kDa

40 kDa

30 kDa

PTEN

260 kDa

140 kDa

100 kDa

MAGI2


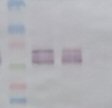


MDA-MB-231

Markers Non- PAR2- IgG

activated activated Isotype


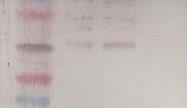


70 kDa

50 kDa

40 kDa

30 kDa


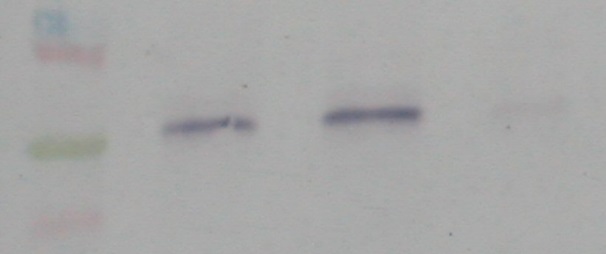


260 kDa

140 kDa

100 kDa

PTEN

MAGI2

Markers PAR2- Non- IgG

activated activated Isotype

LoVo

MDA-MB-231 and LoVo cells (10^3^) were seeded out and sets were incubated with PAR2-AP (20 μM) for up to 30 min. MAGI2 was immunoprecipitated from cell lysates with an anti-MAGI2 (C3; 4 µg) antibody using protein A-magnetic beads. The cell lysates were washed and denatured in SDS-PAGE loading buffer and examined for PTEN and MAGI2 by western blot using a mouse anti-PTEN antibody (217702) and a rabbit anti-MAGI2 antibody.

Supplementary Figure 6. Analysis of the cellular MAGI2 by western blot


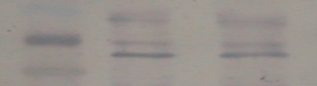

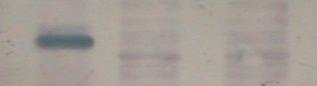


260 kDa

140 kDa

100 kDa

40 kDa

30 kDa

MAGI2

GAPDH

Markers Non- PAR2-

activated activated

Sets of MDA-MB-231 cells (10^3^) were seeded out into 35 mm-glass based μ-dishes and adapted to serum-free medium for 1 h. One set was activated using PAR2-AP (20 µM) for 30 min. The cells were then lysed in Laemmli’s buffer and examined by western blot as described in the methods section. The membranes were blocked, washed and then probed using a rabbit anti-MAGI2 antibody (C3) diluted 1:4000 (v/v) while GAPDH was detected using a rabbit anti-human GAPDH polyclonal antibody, in TBST. The membranes were then washed with TBST and probed with a goat anti-rabbit alkaline phosphatase-conjugated antibody (Santa Cruz), diluted 1:4000 (v/v). Bands were then visualised using the Western Blue stabilised alkaline phosphatase-substrate, recorded and analysed using ImageJ program.
